# Patient reported side-effects of prednisone and methotrexate in a real-world sarcoidosis population

**DOI:** 10.1177/14799731211031935

**Published:** 2021-09-26

**Authors:** Vivienne Kahlmann, Catharina C Moor, Marcel Veltkamp, Marlies S Wijsenbeek

**Affiliations:** 1 Centre of Excellence for Interstitial Lung Diseases and Sarcoidosis, Department of Respiratory Medicine, Erasmus MC, University Medical Center, Rotterdam, The Netherlands; 2 ILD Center of Excellence, Department of Pulmonology, 6028 St. Antonius Hospital, Nieuwegein, The Netherlands; 3 Division of Heart and Lungs, University Medical Center Utrecht, Utrecht, The Netherlands

**Keywords:** Sarcoidosis, quality of life, prednisone, methotrexate, treatment, side-effects

## Abstract

Currently prednisone is the first-line pharmacological treatment option for pulmonary sarcoidosis. Methotrexate is used as second-line therapy and seems to have fewer side-effects. No prospective comparative studies of first-line treatment with methotrexate exist. In this study, we evaluated patient reported presence and bothersomeness of side-effects of prednisone and methotrexate in a sarcoidosis population to guide the design of a larger prospective study. During a yearly patient information meeting 67 patients completed a questionnaire on medication use; 11 patients never used prednisone or methotrexate and were excluded from further analysis. Of the remaining 56 patients, 89% used prednisone and 70% methotrexate (present or former). Significantly more side-effects were reported for prednisone than for methotrexate, 78% versus 49% (*p* = 0.006). In conclusion, methotrexate seems to have fewer and less bothersome side-effects than prednisone. These findings need to be confirmed in a prospective study.

## Background

Sarcoidosis is a granulomatous disease with a heterogeneous presentation. The lungs are involved in over 75% of patients^1^ The decision to start treatment depends on different factors, such as the threat on organ function and symptom severity. Treatment aims at improving organ function, as well as quality of life (QoL).^2^ Prednisone is currently the first-line pharmacological treatment option in pulmonary sarcoidosis. Prednisone leads to short-term improvement of pulmonary function and symptoms.^3^ However, prednisone often has major side-effects, such as weight gain, depression, osteoporosis and diabetes, which may lead to impaired QoL.^4–6^ Hence, patients and doctors report an urgent need for better treatment options. Methotrexate is presently considered second-line therapy and has not been studied as first-line treatment.^3,7^Methotrexate may have fewer side-effects than prednisone. Although the side-effects of prednisone and methotrexate are well known, they have never been compared in patients with sarcoidosis. In this survey, we evaluated patients reported side-effects of prednisone and methotrexate in a real-world sarcoidosis population.

## Methods

During a yearly sarcoidosis patient information meeting at the Erasmus University Medical Center in 2019, patients were invited to complete a 14-item survey on medication use and experiences with prednisone and methotrexate. Patients with confirmed sarcoidosis, treated in the Erasmus Medical Center, were invited by letter to attend the meeting. All patients, who were attending the meeting, were invited to participate. Patients provided informed consent to use the survey data, and could provide additional consent to retrieve information from their medical records. The survey consisted of open-ended questions (Supplementary File 1). If patients used combination therapy and experienced side-effects, they reported to which medication they believed the side-effects belonged. Bothersomeness of side-effects was reported on a scale from 0 (not bothersome at all) to 10 (very bothersome). This study was approved by the Medical Ethical Committee of the Erasmus Medical Center. The chi-square test was used to evaluate between-group differences in the reported side-effects. The Mann–Whitney *U* test was used to compare the number and bothersomeness of side-effects. Data was analyzed in SPSS version 25.0.

## Results

In total, 67 patients completed the questionnaire, 11 patients never used prednisone or methotrexate and were excluded from further analysis. Of the remaining 56 patients, 89% used prednisone, 70% methotrexate and 21% also used other medication (present or former). Mean age was 53 years (SD 9.8) and 57% was female. Of the 41 patients that gave consent to use their clinical data; 98% had hilar or mediastinal lymphadenopathy, 80% had pulmonary involvement, 17% had cardiac involvement, 12% had ocular sarcoidosis, 12% had skin manifestations, 12% had neurological involvement, 5% had bone involvement, 5% had sarcoidosis in the liver, and 5% had renal involvement. Median time after diagnosis was 4 years (IQR 2.2–6.8), median time on medication was 24 months (IQR 6.0–36.0) for prednisone and 12 months (IQR 6.0–24.0) for methotrexate.

**Figure 1.**
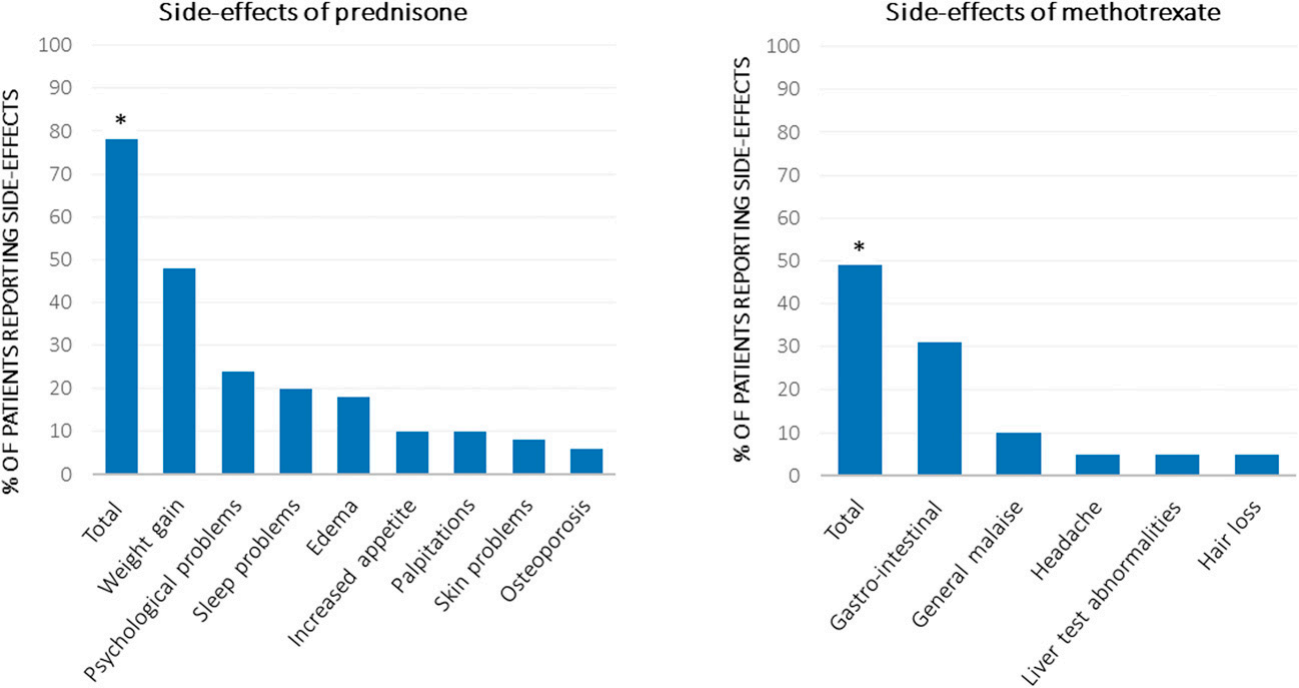
Side-effects reported by 50 patients treated with prednisone and 39 patients treated with methotrexate. Total signifies one or more reported side-effects. ∗Number of patients who reported side-effects was significantly higher for prednisone *p* = 0.006.

Of the 50 patients using prednisone, 78% reported one or more side-effects. Patients most frequently reported weight gain (48%), psychological problems/behavior change (24%), sleep problems/fatigue (20%); results are shown in Figure 1. Median bothersomeness of side-effects (*n* = 20) was 6.0 (IQR 5.0–8.0) and average number of side-effects (*n* = 39) was 2.0 (IQR 1.0–3.0). In the 39 patients treated with methotrexate, fewer side-effects were reported: 49% reported one or more side-effects such as nausea or other gastrointestinal complaints (31%), general malaise (10%), headache (5%), liver test abnormalities (5%) and hair loss (5%). Median bothersomeness of side-effects (*n* = 10) was 4.5 (IQR 3.8–6.2), and average number of side-effects (*n* = 19) was 1.0 (IQR 1.0–2.0).

**Figure 2.**
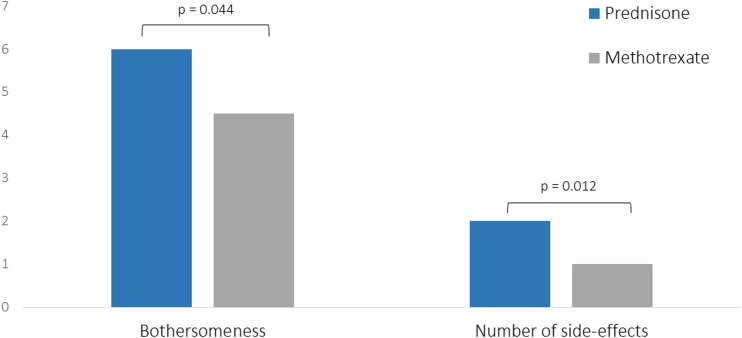
Average number and bothersomeness (on a scale from 0–10) of side-effects of prednisone and methotrexate. Of the 39 patients who experienced side-effects of prednisone, 20 reported bothersomeness of side-effects. Of the 19 patients who experienced side-effects of methotrexate, 10 reported bothersomeness of side-effects.

Significantly more patients with prednisone had side-effects compared with methotrexate (*p* = 0.006). Moreover, patients with prednisone reported a higher number of side-effects (*p* = 0.012) and more bothersome side-effects (*p* = 0.044); results are shown in Figure 2.

## Discussion

This study compared patient reported side-effects of prednisone and methotrexate in a real-world sarcoidosis population. Importantly, patients reported significantly fewer and less bothersome side-effects with methotrexate than with prednisone.

Prednisone is the cornerstone of pharmacological treatment for pulmonary sarcoidosis. Awareness of the potential debilitating side-effects of prednisone and impact on QoL in patients with sarcoidosis has increased.^4^ Hence, the need for steroid-sparing alternatives is mentioned in the most recent consensus statement on treatment of sarcoidosis.^3^ Unfortunately, research into alternative treatment options is scarce. This survey was conducted to explore the rationale for a randomized controlled trial comparing the efficacy and tolerability of methotrexate and prednisone as first-line treatment for sarcoidosis.^8^

Findings of this study indicate that methotrexate seems to have fewer side-effects than prednisone in clinical practice. One of the limitations of this study was that the open-ended questions rely on patients’ recollection. Although recall bias might have had an effect on the reported side-effects, we expect this would be similar in both groups. Furthermore, we focused on patient experiences and therefore information on physician reported side-effects is lacking. As this was a retrospective single-center study, results need to be confirmed in a larger prospective cohort.^8^

In conclusion, the results of this current study reveal the patient perception on prednisone and methotrexate use and emphasize that better evidence-based treatment options with fewer side-effects are highly needed to improve QoL for patients with sarcoidosis.

## Supplemental Material

sj-pdf-1-crd-10.1177_14799731211031935 – Supplemental Material for Patient reported side-effects of prednisone and methotrexate in a real-world sarcoidosis population Click here for additional data file. Supplemental Material, sj-pdf-1-crd-10.1177_14799731211031935 for Patient reported side-effects of prednisone and methotrexate in a real-world sarcoidosis population by Vivienne Kahlmann, Catharina C Moor, Marcel Veltkamp and Marlies S Wijsenbeek in Chronic Respiratory Disease

## References

[1] Thillai M Atkins CP Crawshaw A, et al. BTS clinical statement on pulmonary sarcoidosis. Thorax 2021; 76: 4–20. 33268456 10.1136/thoraxjnl-2019-214348

[2] Wijsenbeek MS Culver DA. Treatment of sarcoidosis. Clin Chest Med 2015; 36: 751–767. 26593147 10.1016/j.ccm.2015.08.015

[3] Rahaghi FF Baughman RP Saketkoo LA, et al. Delphi consensus recommendations for a treatment algorithm in pulmonary sarcoidosis. Eur Respir Rev 2020; 29: 190146. 32198218 10.1183/16000617.0146-2019 PMC9488897

[4] Judson MA Chaudhry H Louis A, et al. The effect of corticosteroids on quality of life in a sarcoidosis clinic: the results of a propensity analysis. Respir Med 2015; 109: 526–531. 25698652 10.1016/j.rmed.2015.01.019 PMC4447298

[5] Khan NA Donatelli CV Tonelli AR, et al. Toxicity risk from glucocorticoids in sarcoidosis patients. Respir Med 2017; 132: 9–14. 29229111 10.1016/j.rmed.2017.09.003

[6] Broos CE Wapenaar M Looman CWN, et al. Daily home spirometry to detect early steroid treatment effects in newly treated pulmonary sarcoidosis. Eur Respir J 2018; 51: 1702089. 29348185 10.1183/13993003.02089-2017

[7] Cremers JP Drent M Bast A, et al. Multinational evidence-based world association of sarcoidosis and other granulomatous disorders recommendations for the use of methotrexate in sarcoidosis: integrating systematic literature research and expert opinion of sarcoidologists worldwide. Curr Opin Pulm Med 2013; 19: 545–561. 23880702 10.1097/MCP.0b013e3283642a7a

[8] Kahlmann V Janssen Bonas M Janssen Bonás M, et al. Design of a randomized controlled trial to evaluate effectiveness of methotrexate versus prednisone as first-line treatment for pulmonary sarcoidosis: the PREDMETH study. BMC Pulm Med 2020; 20: 271. 33076885 10.1186/s12890-020-01290-9 PMC7574228

