# Supplementary material for: Patient reported side-effects of prednisone and methotrexate in a real-world sarcoidosis population
Source: Chron Respir Dis. 2021 Sep 26;18:14799731211031935. doi: 10.1177/14799731211031935 (PMC8477709; doi:10.1177/14799731211031935)
Supplement: sj-pdf-1-crd-10.1177_14799731211031935 – Supplemental Material for Patient reported side-effects of prednisone and methotrexate in a real-world sarcoidosis population [file sj-pdf-1-crd-10.1177_14799731211031935.pdf]

Supplementary file 1: **SURVEY ABOUT MEDICATION USE – SARCOIDOSIS PATIENTDAY**

Age: ..... years **Gender:** male/female

When was the diagnosis sarcoidosis confirmed? *(Example: March 2014)*

……………………………………………………………………

Are you currently using medication for sarcoidosis? Yes/No

If yes, what medication do you use and in what dosage?

……

For how long have you been using the above mentioned medication?

……

Have you experienced any side-effects of your sarcoidosis medication? Yes/No

**Side-effect:**	**Medication?**	**Bothersomeness of side-effects on a scale from 0-10?** *Not bothersome = 0, very bothersome = 10*
………………………………………	………………………………………	………………………………………
………………………………………	………………………………………	………………………………………
………………………………………	………………………………………	………………………………………
………………………………………	………………………………………	………………………………………

Please fill in the next three questions if you have used prednisone in the past

Did you experience side-effects of prednisone? If yes, please describe the most important side-effects.

……

Why did you stop taking prednisone? *(example: side-effects, prednisone was not effective)*

……

How long did you use prednisone?

……

Please fill in the next three questions if you have used methotrexate in the past

Did you experience side-effects of methotrexate? If yes, please describe the most important side-effects.

……

Why did you stop taking methotrexate? *(example: side-effects, methotrexate was not effective)*

……

How long did you use methotrexate?

……
